# Intercellular genetic tracing by alternative synthetic Notch signaling

**DOI:** 10.1038/s41421-024-00721-9

**Published:** 2024-10-15

**Authors:** Kuo Liu, Shaohua Zhang, Xinfeng Meng, Hongxin Li, Jingting Zhu, Enci Wang, Muxue Tang, Mingjun Zhang, Bin Zhou, Lixin Wang

**Affiliations:** 1 Key Laboratory of Systems Health Science of Zhejiang Province, School of Life Science, Hangzhou Institute for Advanced Study, University of Chinese Academy of Sciences, Hangzhou, Zhejiang China; 2 New Cornerstone Investigator Institute, Key Laboratory of Multi-Cell Systems, Shanghai Institute of Biochemistry and Cell Biology, Center for Excellence in Molecular Cell Science, Chinese Academy of Sciences, Shanghai, China; 3 School of Life Science and Technology, ShanghaiTech University, Shanghai, China; 4 Department of Vascular Surgery, Zhongshan Hospital, Fudan University, Shanghai, China; 5 Xiamen Municipal Vascular Disease Precise Diagnose & Treatment Lab, Xiamen, Fujian China

**Keywords:** Cell migration, Stem-cell niche

Dear Editor,

Genetic tools for tracking in vivo cell‒cell communication are crucial for analyzing cellular behaviors and their regulatory mechanisms. Many new techniques have been recently developed to label neighboring cells and to study cell‒cell interactions^1–13^. Recently, a synthetic Notch (synNotch)-based intercellular genetic system called genetic tracing of cell‒cell contact (gTCCC) was developed. This system utilizes artificial ligands and receptors specific to sender and receiver cells, enabling successful genetic recording of cell‒cell contact in vivo^1,2,9,10^. However, the applicability of alternative synNotch versions for tracing cell‒cell contact in vivo has yet to be demonstrated to broaden its diverse application in multiple biological processes. Considering the complexity of cellular communication in multicellular organisms, it is important to construct diverse gTCCC systems for investigations of multiple complex cellular behaviors in vivo.

In this study, we constructed new intercellular genetic systems based on two alternative synNotch systems. In the first system, sender cells express a synNotch ligand by replacing the extracellular domain of Notch ligand with a membrane-tethered CD19 protein (mCD19), while receiver cells express a synNotch receptor with its extracellular and intracellular domains replaced by Myc-tagged anti-CD19 nanobody (αCD19) and tetracycline (tet) trans-activator (tTA), respectively (Fig. 1a)^1,2^. The transmembrane domain of the synNotch receptor remains intact for γ-secretase recognition. Expression of the synNotch ligand and receptor is driven by different promoters in sender and receiver cells. To differentiate it from our previous technology^9,10^, we referred to the mice carrying these elements as gTCCC2 mice, denoting the second version of “genetic tracing of cell‒cell contact”. Theoretically, when sender and receiver cells interact, the synNotch ligand binds to the receptor, leading to the release and binding of tTA on the tet promoter in the nucleus, thereby the receiver cells can be genetically labeled by the reporter (Fig. 1b). We then constructed two sender mouse lines, *Rosa26-loxP-Stop-loxP-mCD19* (*R26-L-mCD19*) mice and *Rosa26-rox-Stop-rox-mCD19* (*R26-R-mCD19*) mice, which allows mCD19 expression under the control of cell type-specific Cre or Dre lines (Fig. 1c and Supplementary Fig. S1a, b). We also constructed a receiver mouse line, *Cdh5-αCD19NtTA-tdT*, which enables studying CDH5^+^ endothelial cells (ECs) labeled by cell contact (Fig. 1c and Supplementary Fig. S1c). We confirmed that in *Cdh5-αCD19NtTA-tdT* mice, there was no tdT expression and all CDH5^+^ ECs expressed the Myc-tag (Fig. 1d, e). In *ACTB-Cre;Cdh5-αCD19NtTA-tdT* mice, all ECs were labeled with tdT and they no longer expressed the Myc-tag after Cre-loxP recombination (Fig. 1d, e).

**Fig. 1:**
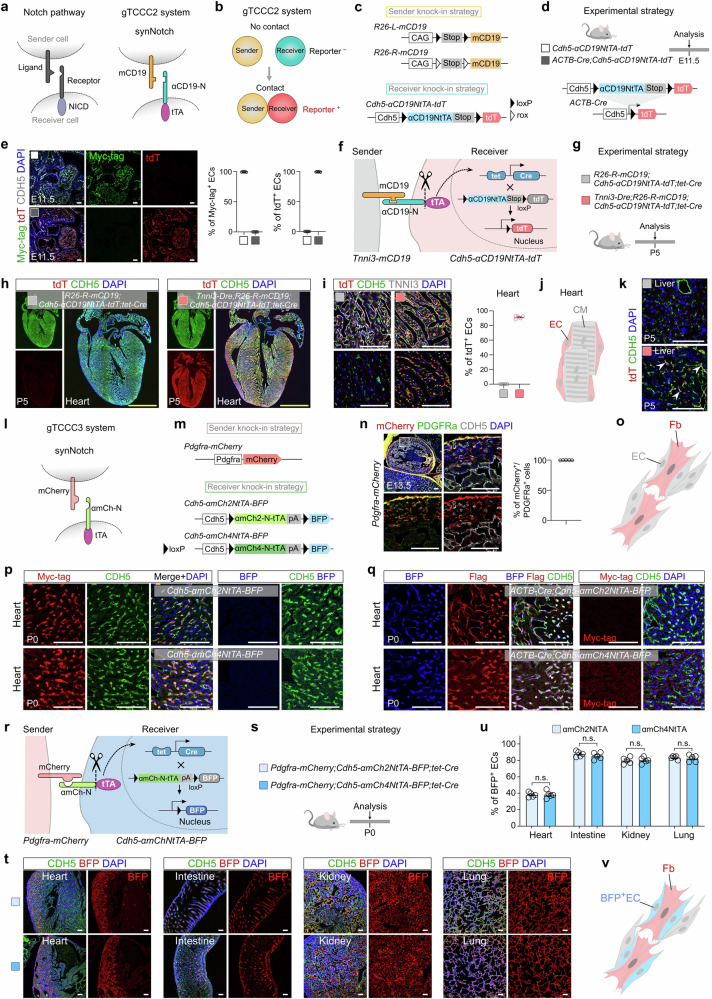
Generation of intercellular genetic systems based on CD19 or mCherry for tracing cell‒cell contact in vivo. **a** Schematic diagrams showing the canonical Notch pathway and the synNotch pathway. **b** A schematic diagram showing the gTCCC2 system for genetic tracing of cell‒cell contact. **c** A schematic diagram showing knockin strategies for *R26-L-mCD19*, *R26-R-mCD19*, and *Cdh5-αCD19NtTA-tdT* line. **d** A schematic diagram showing the experimental design. **e** Immunostaining for Myc-tag, tdT, and CDH5 on E11.5 embryonic sections in **d** (left panel). Quantification of the percentage of ECs expressing Myc-tag or tdT (right panel). Data are presented as means ± SEM; *n* = 5. **f** A schematic diagram showing the gTCCC2 system for genetic tracing of CDH5^+^ ECs that have contact with TNNI3^+^ cells. **g** A schematic diagram showing the experimental design. **h**, **i** Sectional immunostaining for tdT, CDH5, and TNNI3 on P5 heart sections. Quantification of the percentage of ECs expressing tdT (**i**, right panel). Data are presented as means ± SEM; *n* = 5. **j** A cartoon image showing the ECs adjacent to cardiomyocytes (CM) are labeled by tdT. **k** Sectional immunostaining for tdT and CDH5 on P5 liver sections. White arrowheads, tdT^+^CDH5^+^ ECs. **l** A schematic diagram showing the synNotch pathway of gTCCC3 system. **m** The knockin strategies of *Pdgfra-mCherry*, *Cdh5-αmCh2NtTA-BFP*, and *Cdh5-αmCh4NtTA-BFP* mice. **n** Immunostaining for mCherry, PDGFRa, and CDH5 on E13.5 embryonic sections. Quantification of the percentage of the PDGFRa^+^ fibroblasts expressing mCherry. **o** A cartoon image showing the EC is distributed adjacent to fibroblast (Fb). **p** Immunostaining for Myc-tag, CDH5, and BFP on P0 heart sections. **q** Immunostaining for BFP, Flag, and CDH5 on P0 heart sections. **r** A schematic diagram showing the gTCCC3 system for genetic tracing of CDH5^+^ ECs that have contact with PDGFRa^+^ cells. **s** A schematic diagram showing the experimental design. **t** Immunostaining for BFP and CDH5 on P0 tissue sections. **u** Quantification of the percentage of ECs expressing BFP in **t**. Data are means ± SEM; *n* = 5. *P* > 0.05. n.s., not significant. **v** A cartoon image showing the ECs adjacent to Fb are labeled by BFP. Scale bars: white, 100 µm; yellow, 1 mm.

Next, we employed this system to investigate the contact between cardiomyocytes and ECs in the heart. In *Tnni3-Dre;R26-R-mCD19;Cdh5-αCD19NtTA-tdT;tet-Cre* quadruple-positive (gTCCC2) mice, Tnni3^+^ cardiomyocytes express the synNotch ligand mCD19. Upon contact between cardiomyocytes and ECs, tTA is released and binds to the tet promoter, leading to Cre expression and subsequent genetic tracing of the cardiomyocyte-contacting ECs as tdT after Cre-loxP recombination (Fig. 1f, g). The *R26-R-mCD19;Cdh5-αCD19NtTA-tdT;tet-Cre* triple-positive mice lacking synNotch ligand expression were used as the control group (Fig. 1f, g). Immunostaining analysis demonstrated specific labeling of ECs adjacent to cardiomyocytes in the gTCCC2 mice, while ECs were not labeled in the control mice, confirming the successful recording of cardiomyocyte‒EC interactions (Fig. 1h‒j and Supplementary Fig. S1d‒k). We also detected a subset of tdT^+^ ECs in the postnatal liver (Fig. 1k and Supplementary Fig. S1l), which is consistent with our previous studies^9,14^.

Next, we developed another synNotch system utilizing anti-mCherry nanobodies^15^. In this system, we replaced the extracellular domain of the synNotch ligand with membrane-tethered mCherry protein (mCherry), and the extracellular and intracellular domains were replaced by Myc-tagged anti-mCherry nanobody (αmCherry) and tTA, respectively (Fig. 1l). The binding of mCherry to αmCherry could theoretically be used for triggering synNotch system to genetic trace cell‒cell contact. We referred to the mice carrying these synthetic elements as gTCCC3 mice. We generated a new sender line, *Pdgfra-mCherry*, where Pdgfra^+^ fibroblasts served as the sender cells, and two versions of endothelial receiver lines, *Cdh5-αmCh2NtTA-BFP* and *Cdh5-αmCh4NtTA-BFP*, which contained different anti-mCherry nanobodies (αmCherry2 and αmCherry4) with low and high affinity^15^, respectively, to mark the receiver cells (Fig. 1m and Supplementary Fig. S2a‒c). Our data demonstrated that all PDGFRa^+^ fibroblasts in *Pdgfra-mCherry* mice were labeled with mCherry, and a subset of mCherry^+^ cells were in close position with ECs (Fig. 1n, o). Furthermore, characterization of the receiver lines revealed Myc-tag expression in ECs without BFP leakiness (Fig. 1p and Supplementary Fig. S2d‒f). However, upon *ACTB-Cre*-mediated Cre-loxP recombination, all ECs expressed BFP with no longer expression of Myc-tag (Fig. 1q and Supplementary Fig. S3).

Considering the proximity of fibroblasts and ECs, we investigated their direct communication in vivo. In the gTCCC3 system, when ECs had contact with fibroblasts, the tTA is released to induce tet-Cre expression, resulting in BFP labeling of ECs after Cre-loxP recombination (Fig. 1r, s). Analysis of P0 tissues confirmed BFP labeling in a subset of ECs present in multiple organs, indicating the potential of this system for studying fibroblast‒EC contact in vivo (Fig. 1t‒v and Supplementary Fig. S4a‒f). Quantification analysis revealed no significant difference in the labeling efficiency of ECs between αmCherry2- and αmCherry4-containing receivers (Fig. 1u). The majority of ECs in most organs were randomly labeled with BFP, whereas, in the liver and brain, this labeling was observed in about 35% and minimal populations, respectively (Supplementary Fig. S4c‒f). Notably, an enrichment of BFP^+^ ECs was observed in the myocardial compaction layer and atria of the heart. Additionally, a subpopulation of valvular ECs expressing Prox1 was labeled at the outflow side (fibrosa) of the aortic valves (Supplementary Fig. S4c, d). Further analysis of P9 tissues revealed an increased proportion of BFP-labeled ECs in most organs, whereas minimal labeling persisted in the brain (Supplementary Fig. S5a‒d). These data suggest that contact between ECs and fibroblasts occurs continuously after birth. We further explored the real-time interaction between ECs and fibroblasts by using *Pdgfra-mCherry;Cdh5-αmCh2NtTA-BFP;tet-tdT* mice, which is named as gLCCC system for “genetic labeling of cell‒cell contact” (Supplementary Fig. S6a)^9^. We showed that 11.11 ± 0.70% of ECs in the heart and 2.86 ± 0.46% of ECs in the liver were labeled with tdT, whereas tdT^+^ ECs were barely detectable in other organs (Supplementary Fig. S6b‒f). Interestingly, we further identified that the Prox1^+^ valvular VECs still maintained their real-time contact with the valvular fibroblasts (Supplementary Fig. S6c).

We then used the cardiomyocytes‒ECs interaction model to test the orthogonal nature of gTCCC, gTCCC2, and gTCCC3 systems in vivo (Supplementary Fig. S7a). We designed three distinct types of adeno-associated viruses (AAVs) to induce cardiomyocytes to express unique synNotch ligands: mGFP, Myc-tagged-mCD19, or mCherry. We first injected these AAVs into *Cdh5-αGFPNtTA-tdT;tetO-BFP-Dre* mice to monitor cardiomyocytes‒ECs contact. We revealed that the ECs were specifically labeled in the cardiomyocytes-mGFP group, whereas no such labeling was detected in the cardiomyocytes-mCD19 or cardiomyocytes-mCherry groups (Supplementary Fig. S7b‒g). Furthermore, the injection of AAVs into alternative genetic tracing mouse models, namely *Cdh5-aGFPtTA;tet-Cre;R26-tdT* and *Cdh5-aGFPtTA;tet-Cre;R26-GFP*, corroborated these findings (Supplementary Fig. S8).

In summary, we develop two intercellular genetic systems, gTCCC2 and gTCCC3 for genetic tracing of cell‒cell contact in vivo. These systems can be combined with the Cre-loxP system for specific gene manipulation in contacting cells. They can be used orthogonally with our previously reported synNotch system to provide a versatile genetic platform for studying complex cell‒cell behaviors and communications in vivo. Moreover, further investigation is required to determine if fibroblasts‒EC interaction is critical for early organogenesis, especially cardiac and valvular development.

## Supplementary information

Supplementary Information

## Data Availability

All study data are included in the article and/or supplementary information.
