# Supplementary material for: Intercellular genetic tracing by alternative synthetic Notch signaling
Source: Cell Discov. 2024 Oct 15;10:101. doi: 10.1038/s41421-024-00721-9 (PMC11480435; doi:10.1038/s41421-024-00721-9)
Supplement: Supplementary file 1 — Supplementary Information [file 41421_2024_721_MOESM1_ESM.pdf]

Supplementary Information for

Intercellular genetic tracing by alternative synthetic Notch signaling

Kuo Liu, Shaohua Zhang, Xinfeng Meng, Hongxin Li, Jingting Zhu, Enci Wang, Muxue Tang, Mingjun Zhang, Bin Zhou*, Lixin Wang*

*Corresponding author: Bin Zhou, Lixin Wang

Email: zhoubin@sibs.ac.cn (B.Z.); wang.lixin@zs-hospital.sh.cn (L.W.)

This PDF file includes:

Supplementary Methods

Supplementary Figs. S1-S8

Supplementary Figure Legends

Methods

Mice

All mouse experiments in this study were strictly within the Institutional Animal Care and Use Committee (IACUC) guidelines of the Institute of Biochemistry and Cell Biology, Shanghai Institutes for Biological Sciences, Chinese Academy of Sciences. mouse lines were described previously. The *Cdh5-αGFPNtTA-tdT*, *tet-Cre*, *tet-tdT*, *tet-Dre-BFP*, *Rosa26-LSL-tdTomato* (*R26-tdT*), and *Rosa26-LSL-GFP* (*R26-GFP*) line were described previously[1, 2]. The *Rosa26-loxP-stop-loxP-mCD19* (*R26-L-mCD19*) mice line was generated by inserting the CAG-loxP-Stop-loxP-mCD19 component between exon1 and exon2 of *Rosa26* gene locus through homologous recombination. The *Rosa26-rox-Stop-rox-mCD19* (*R26-R-mCD19*) mice line was generated by inserting the CAG-rox-stop-rox-mCD19 component between exon1 and exon2 of *Rosa26* gene locus through homologous recombination. The *Cdh5-αCD19NtTA-tdT* mice line was generated by inserting the loxP-myc-αCD19-N-tTA-Stop-loxP-tdTomato component before the exon2 of endogenous *Cdh5* gene locus via homologous recombination. The *Pdgfra-mCherry* mice line was generated by inserting the mCherry sequence before the exon2 of the endogenous *Pdgfra* gene locus via homologous recombination. The *Cdh5-αmCh2NtTA-BFP* mice line was generated by knocking the loxP-αmCh2-N-tTA-pA-loxP-BFP sequence before the exon2 of endogenous *Cdh5* gene locus via homologous recombination. The *Cdh5-αmCh4NtTA-BFP* mice line was generated by knocking the loxP-αmCh4-N-tTA-pA-loxP-BFP sequence before the exon2 of endogenous Cdh5 gene locus via homologous recombination. *R26-L-mCD19*, *R26-R-mCD19*, *Pdgfra-mCherry*, *Cdh5-αmCh2NtTA-BFP*, and *Cdh5-αmCh4NtTA-BFP* mouse lines were generated by Shanghai Model Organisms Center, Inc (SMOC). All mice used in this study were kept at C57BL6/ICR mixed backgrounds.

Genomic PCR

Genomic DNA was prepared from mouse tails, litters, or embryonic yolk sacs. In Brief, the tissues were digested by lysis buffer (100 mM Tris–HCl, PH 7.8, 5 mM EDTA, 0.2% SDS, 200 mM NaCl) with protease K at 55 °C overnight. Then the genomic DNA was

precipitated with isopropanol and the supernatant was discarded after maximum centrifugation. The DNA was washed with 70% ethanol and the supernatant was discarded. 200 μ l of distilled water was added to dissolve the DNA after air-frying for PCR amplification. All of the embryos and mice were genotyped using genomic PCR as described previously.

AAV production and treatment

The AAVs were produced with cis-plasmids containing the full cardiac TNNT2 promoter, synNotch ligands, and followed WPRE-pA cassette was under the control of TNNT2 promoter. The three types of virus AAV2/9-TNNT2-mGFP-WPRE-pA (AAV9-TNNT2-mGFP), AAV2/9-TNNT2-mCD19-Myc-tag-WPRE-pA (AAV9-TNNT2-mCD19), and AAV2/9-TNNT2-mCherry-WPRE-pA (AAV9-TNNT2-mCherry) were packaged in HEK293T cells. Viral particles were harvested three days post-transfection and purified by Taitool Bioscience (Shanghai, China). The titers of the vector genome were quantified via qPCR using vector-specific primers. For *in vivo* inducing cardiomyocytes expression of synNotch ligands, the neonatal P1 mice were injected subcutaneously at $1.5 \sim 2.0 \times 10^{11}$ titer virus on their backs and then the hearts were collected for analysis at P9.

Tissue collection and immunofluorescent staining

After euthanizing the mice, the embryos or other tissues were fixed in 4% PFA (paraformaldehyde) at 4 °C for 20 min to 1 hour according to the organ size and then washed several times in PBS. After that, the tissues were placed on 1% agar gel for whole-mount bright-field and fluorescence images using a Zeiss stereoscope (Axio Zoom.V16). The tissues were dehydrated in a 30% sucrose in PBS solution overnight at 4 °C, and then embedded in optimum cutting tissue (O.C.T., Sakura) and stored at −80 °C until sectioning. Sections (10 μm) were cut from embedded blocks and then collected on slides.

For immunostaining, the sections were air-dried before immunostaining and then washed three times in PBS. Subsequently, the slides were blocked with PBSST (0.1%

Triton X-100 and 2.5% normal donkey serum in PBS) for 30 min at room temperature and incubated with the primary antibody overnight at 4 °C. After that, the sections were washed 3 times in PBS and then incubated with secondary antibodies and DAPI for nuclear staining at room temperature for 40 min in dark. The tissue sections were mounted with the mounting medium after washing in PBS for next confocal analysis. For weak signals, horseradish peroxidase-conjugated antibodies were used to amplify the signals. Images from immunostained samples were acquired by using an Olympus confocal microscope (FV1200), and the obtained images were analyzed by ImageJ (NIH) software.

Primary antibodies to the following proteins were used:

tdTomato (rabbit, 600-401-379; 1:1,000 dilution; Rockland), tdTomato (goat, 200-101-379; 1:1,000 dilution; Rockland), PDGFRa (goat, AF1062, 1:500 dilution; R&D), Myc-tag (rabbit, 2272, 1:500 dilution; Cell signaling), Troponin I (goat, ab56357, 1:200 dilution; Abcam). BFP (rabbit, AB233, 1:100 dilution; Evrogen), Flag (mouse, F1804, 1:100 dilution; Sigma), CDH5/VE-cad (Goat, AF1002, 1:200 dilution; R&D), Prox1 (goat, AF2727, 1:100 dilution; R&D), aSMA (mouse, F3777, 1:500 dilution; Sigma).

Alexa fluorescence conjugated secondary antibodies used in this study: Alexa donkey anti-rabbit 488 (donkey, A21206, 1:1,000; Invitrogen), Alexa donkey anti-rabbit 555 (donkey, A31572, 1:1,000; Invitrogen), Alexa donkey anti-rabbit 647 (donkey, A31573, 1:1,000; Invitrogen), donkey anti-goat 488 (donkey, A11055, 1:1,000; Invitrogen), donkey anti-goat 555 (donkey, A21432, 1:1,000; Invitrogen), donkey anti-goat 647 (donkey, A21447, 1:1,000; Invitrogen).

Statistics analysis

All data were obtained from five independent experiments as indicated in figure legends and presented as mean values ± s.e.m. Two-tailed unpaired Student's t-test was used for statistical comparison between two groups. $P < 0.05$ was accepted as statistically significant.

References

1 Zhang S, Zhao H, Liu Z *et al.* Monitoring of cell-cell communication and contact history in mammals. *Science* 2022; **378**:eabo5503.

2 He L, Pu W, Liu X *et al.* Proliferation tracing reveals regional hepatocyte generation in liver homeostasis and repair. *Science* 2021; **371**.

Supplementary figure legends:

Supplementary Figure 1 | Generation of *R26-L-mCD19*, *R26-R-mCD19*, and *Cdh5-αCD19NtTA-tdT* mice.

a, A schematic diagram showing the knock-in strategy of *R26-L-mCD19* by homologous recombination. **b**, A schematic diagram showing the knock-in strategy of *R26-R-mCD19* by homologous recombination. **c**, A schematic diagram showing the knock-in strategy of *Cdh5-αCD19NtTA-tdT* by homologous recombination. **d**, A schematic diagram showing the experimental design. The *Tnni3-Dre;Cdh5-αCD19NtTA-tdT;tet-Cre* mice were set as the control group, which lacks the genotype, *R26-R-mCD19*, in comparison to the experimental group of *Tnni3-Dre;R26-R-mCD19;Cdh5-αCD19NtTA-tdT;tet-Cre* quadruple positive mice in Fig. 1g. **e**, Whole-mount fluorescence view of postnatal 5 (P5) organs in (**d**). **f**, Immunostaining for tdT and CDH5 on P5 tissue sections of the *Tnni3-Dre;Cdh5-αCD19NtTA-tdT;tet-Cre* triple-positive mice. **g**, A schematic diagram showing the experimental design. The *Tnni3-Dre;R26-R-mCD19;Cdh5-αCD19NtTA-tdT* mice were set as the control group, which lacks the genotype, *tet-Cre*, in comparison to the experimental group of *Tnni3-Dre;R26-R-mCD19;Cdh5-αCD19NtTA-tdT;tet-Cre* quadruple positive mice in Fig. 1g. **h**, Whole-mount fluorescence view of postnatal 5 (P5) organs in (**g**). **i**, Immunostaining for tdT and CDH5 on P5 tissue sections of the *Tnni3-Dre;R26-R-mCD19;Cdh5-αCD19NtTA-tdT* triple-positive mice. **j**, A schematic diagram showing the experimental design, which was the same as Fig. 1g. **k**, Whole-mount fluorescence view of P5 hearts

in (**j**). **l**, Whole-mount fluorescence view of P5 livers in (**j**) Scale bars, white bars, 100 μm; yellow bars, 1 mm.

Supplementary Figure 2 | Generation and characterization of *Pdgfra-mCherry*, *Cdh5-αmCh2NtTA-BFP*, and *Cdh5-αmCh4NtTA-BFP* mice.

a, A schematic diagram showing the knock-in strategy of *Pdgfra-mCherry* by homologous recombination. **b**, A schematic diagram showing the knock-in strategy of *Cdh5-αmCh2NtTA-BFP* by homologous recombination. **c**, A schematic diagram showing the knock-in strategy of *Cdh5-αmCh4NtTA-BFP* by homologous recombination. **d**, A schematic diagram showing the experimental design. **e**, A schematic showing the components of αmCh2-N-tTA and αmCh4-N-tTA could express while the BFP would not express because of the existence of poly A (pA). **f**, Immunostaining for Myc-tag, CDH5, and BFP on P0 tissue sections of *Cdh5-αmCh2NtTA-BFP* and *Cdh5-αmCh4NtTA-BFP* mice. Scale bars, 100 μm.

Supplementary Figure 3 | Characterization of *Cdh5-αmCh2NtTA-BFP* and *Cdh5-αmCh4NtTA-BFP* mice.

a, A schematic diagram showing the experimental design. After Cre-loxP recombination, the component of αmCh2-N-tTA-pA would be removed and the BFP would be expressed. **b**, Immunostaining for BFP, Flag, and CDH5 on P0 tissue sections of *ACTB-Cre;Cdh5-αmCh2NtTA-BFP* mice. **c**, A schematic diagram showing the experimental design. After Cre-loxP recombination, the component of αmCh4-N-tTA-pA would be removed and the BFP would be expressed. **d**, Immunostaining for BFP, Flag, and CDH5 on P0 tissue sections of *ACTB-Cre;Cdh5-αmCh4NtTA-BFP* mice. Scale bars, 100 μm.

Supplementary Figure 4 | Characterization of the endothelial cells that have contacted with fibroblasts in P0 *Pdgfra-mCherry;Cdh5-αmCh2NtTA-BFP;tet-Cre*

mice.

a, A schematic diagram showing the fibroblasts-contacted endothelial cells could be permanently labeled as BFP after Cre-loxP recombination by gTCCC3 system. **b**, A schematic diagram showing the experimental design. The P0 tissues of *Pdgfra-mCherry;Cdh5-αmCh2NtTA-BFP;tet-Cre* mice were collected for analysis. **c**, Immunostaining for BFP and CDH5 on P0 heart sections of *Pdgfra-mCherry;Cdh5-αmCh2NtTA-BFP;tet-Cre* mice. Yellow arrowheads, $BFP^{+}CDH5^{+}$ cells. **d**, Immunostaining for BFP, Prox1, aSMA, and CDH5 on P0 heart sections of *Pdgfra-mCherry;Cdh5-αmCh2NtTA-BFP;tet-Cre* mice. Yellow arrowheads, $BFP^{+}Prox1^{+}$ cells or $BFP^{+}CDH5^{+}$ cells. **e**, Immunostaining for BFP and CDH5 on P0 tissues sections of *Pdgfra-mCherry;Cdh5-αmCh2NtTA-BFP;tet-Cre* mice. Yellow arrowheads, $BFP^{+}CDH5^{+}$ cells. **f,** Quantification of the percentage of the $CDH5^{+}$ endothelial cells expressing BFP. Data are the mean ± SEM; n = 5. RA, right atrium. RV, right ventricle. VS, interventricular septum. LV, left ventricle. Scale bars, 100 μm.

Supplementary Figure 5 | Characterization of the endothelial cells that have contacted with fibroblasts in P9 *Pdgfra-mCherry;Cdh5-αmCh2NtTA-BFP;tet-Cre* mice.

a, A schematic diagram showing the experimental design. The P9 tissues of *Pdgfra-mCherry;Cdh5-αmCh2NtTA-BFP;tet-Cre* mice were collected for analysis. **b**, Immunostaining for BFP and CDH5 on P9 heart sections of *Pdgfra-mCherry;Cdh5-αmCh2NtTA-BFP;tet-Cre* mice. Yellow arrowheads, $BFP^{+}CDH5^{+}$ cells. **c**, Immunostaining for BFP and CDH5 on P9 tissues sections of *Pdgfra-mCherry;Cdh5-αmCh2NtTA-BFP;tet-Cre* mice. Yellow arrowheads, $BFP^{+}CDH5^{+}$ cells. **d,** Quantification of the percentage of the $CDH5^{+}$ endothelial cells expressing BFP. Data are the mean ± SEM; n = 5. RV, right ventricle. VS, interventricular septum. LV, left ventricle. Scale bars, 100 μm.

Supplementary Figure 6 | Characterization of the endothelial cells that contacting with fibroblasts in P0 *Pdgfra-mCherry;Cdh5-αmCh2NtTA-BFP;tet-tdT* mice.

a, A schematic diagram showing the fibroblasts-contacting endothelial cells could be real-time labeled as tdT. The endothelial cells will not be labeled if lost the contact with fibroblasts. **b**, A schematic diagram showing the experimental design. The P0 tissues of *Pdgfra-mCherry;Cdh5-αmCh2NtTA-BFP;tet-tdT* mice were collected for analysis. **c**, Immunostaining for tdT and CDH5 on P0 heart sections of *Pdgfra-mCherry;Cdh5-αmCh2NtTA-BFP;tet-tdT* mice. Since the tdT primary antibody recognizes both the tdT and mCherry antigens, the red signal indicates both the tdT and mCherry signals (tdT+mCherry). Yellow arrowheads, $tdT^{+}CDH5^{+}$ cells. White arrowheads, $tdT^{-}CDH5^{+}$ cells. **d**, Immunostaining for tdT and Prox1 on P0 heart sections of *Pdgfra-mCherry;Cdh5-αmCh2NtTA-BFP;tet-tdT* mice. White arrowheads, $tdT^{-}Prox1^{+}$ lymphatic endothelial cells. Yellow arrowheads, $tdT^{+}Prox1^{+}$ valvular endothelial cells. **e**, Immunostaining for tdT and CDH5 on P0 tissues sections of *Pdgfra-mCherry;Cdh5-αmCh2NtTA-BFP;tet-tdT* mice. White arrowheads, $BFP^{-}CDH5^{+}$ cells. **f,** Quantification of the percentage of the $CDH5^{+}$ endothelial cells expressing tdT. Data are the mean ± SEM; n = 5. RA, right atrium. RV, right ventricle. VS, interventricular septum. LA, left atrium. LV, left ventricle.Scale bars, 100 μm.

Supplementary Figure 7 | SynNotchs of mGFP, mCD19, and mCherry could be orthogonal used *in vivo*.

a, The left schematic diagram showing the $mGFP^{+}$ sender cells to label the contacting endothelial cells as BFP by utilizing the synNotchs of mGFP/anti-mGFP of the gTCCC system. The right schematic diagram showing the well-established cardiomyocyte (CM)-endothelial cell (EC) contact model. Fb, fibroblast. **b**, A schematic diagram showing the experimental design. Three adeno-associated viruses (AAV) that induced the specific expression of synNotch ligands in cardiomyocytes were constructed, namely AAV2/9-TNNT2-mGFP-WPRE-pA (AAV9-TNNT2-mGFP), AAV2/9-TNNT2-mCD19-Myc-tag-WPRE-pA (AAV9-TNNT2-mCD19), and AAV2/9-

TNNT2-mCherry-WPRE-pA (AAV9-TNNT2-mCherry), respectively. The AAV2/9-TNNT2-mGFP was set as positive control. These three AAVs were injected at P1 of *Cdh5-αGFPNtTA;tet-Dre-BFP* mice, respectively, then the hearts were collected for analysis 8 days later. **c**, Immunostaining for mGFP and cardiomyocytes marker TNNI3 and quantification of the percentage of TNNI3 expressing mGFP on P9 heart sections after AAV9-TNNT2-mGFP injection (left panel). Immunostaining for Myc-tag and TNNI3 and quantification of the percentage of TNNI3 expressing Myc-tag on P9 heart sections after AAV9-TNNT2-mCD19 injection (middle panel). Immunostaining for mCherry and TNNI3 and quantification of the percentage of TNNI3 expressing mCherry on P9 heart sections after AAV9-TNNT2-mCherry injection (right panel). Data are the mean ± SEM; n = 5. **d,** Whole-mount fluorescence views of the hearts of three groups after AAV injection in (b). **e,** Immunostaining for mGFP, BFP, and CDH5 on P9 heart sections showed that almost all cardiac endothelial cells were labeled by BFP after AAV9-TNNT2-mGFP injection (left panel). Quantification of the percentage of the $CDH5^{+}$ endothelial cells expressing BFP (middle panel). Data are the mean ± SEM; n = 5. A cartoon image showing the synNotch receptor of anti-mGFP specific recognize the synNotch ligand of mGFP. **f,** Immunostaining for Myc-tag, BFP, and CDH5 on P9 heart sections showed that detected no $BFP^{+}CDH5^{+}$ cells after AAV9-TNNT2-mCD19 injection (left panel). Quantification of the percentage of the $CDH5^{+}$ endothelial cells expressing BFP (middle panel). Data are the mean ± SEM; n = 5. A cartoon image showing the anti-mGFP could not recognize mCD19 ligand. **g,** Immunostaining for mCherry, BFP, and CDH5 on P9 heart sections showed that no $BFP^{+}CDH5^{+}$ cells were detected after AAV9-TNNT2-mCherry injection (left panel). Since the BFP primary antibody recognizes both the BFP and mCherry antigens, the green signal indicates both the BFP and mCherry signals. Quantification of the percentage of the $CDH5^{+}$ endothelial cells expressing BFP (middle panel). Data are the mean ± SEM; n = 5. A cartoon image showing the anti-mGFP could not recognize mCherry ligand. Scale bars, 100 μm.

Supplementary Figure 8 | SynNotchs of mGFP, mCD19, and mCherry have no cross talk *in vivo*.

a, A schematic diagram showing the endothelial cells that have contacted with $mGFP^+$ sender cells could be permanently labeled in the gTCCC system. **b**, A schematic diagram showing the experimental design. The AAV9-TNNT2-mGFP and AAV9-TNNT2-mCD19 were injected at P1 of *Cdh5-αGFPNtTA;tet-Cre;R26-tdT* mice, respectively, then the hearts were collected for analysis after 8 days. The AAV9-TNNT2-mCherry were injected at P1 of *Cdh5-αGFPNtTA;tet-Cre;R26-GFP* mice, then the hearts were collected for analysis after 8 days. **c**, Whole-mount fluorescence views of the hearts after injection of AAV9-TNNT2-mGFP (left panel). Immunostaining for mGFP, tdT, and CDH5 on P9 heart sections showed that almost all cardiac endothelial cells were labeled by tdT after AAV9-TNNT2-mGFP injection (middle panel). Quantification of the percentage of the $CDH5^+$ endothelial cells expressing tdT (right panel). Data are the mean ± SEM; n = 5. **d**, Whole-mount fluorescence views of the hearts after injection of AAV9-TNNT2-mCD19 (left panel). Immunostaining for Myc-tag, tdT, and CDH5 on P9 heart sections showed that no tdT^+CDH5^+ cells were detected after AAV9-TNNT2-mCD19 injection (middle panel). Quantification of the percentage of the $CDH5^+$ endothelial cells expressing tdT (right panel). Data are the mean ± SEM; n = 5. **e**, Whole-mount fluorescence views of the hearts after injection of AAV2/9-TNNT2-mCherry (left panel). Immunostaining for mCherry, GFP, and CDH5 on P9 heart sections showed that no GFP^+CDH5^+ cells were detected after AAV9-TNNT2-mCherry injection (middle panel). Quantification of the percentage of the $CDH5^+$ endothelial cells expressing GFP (right panel). Data are the mean ± SEM; n = 5. Scale bars, 100 μm.

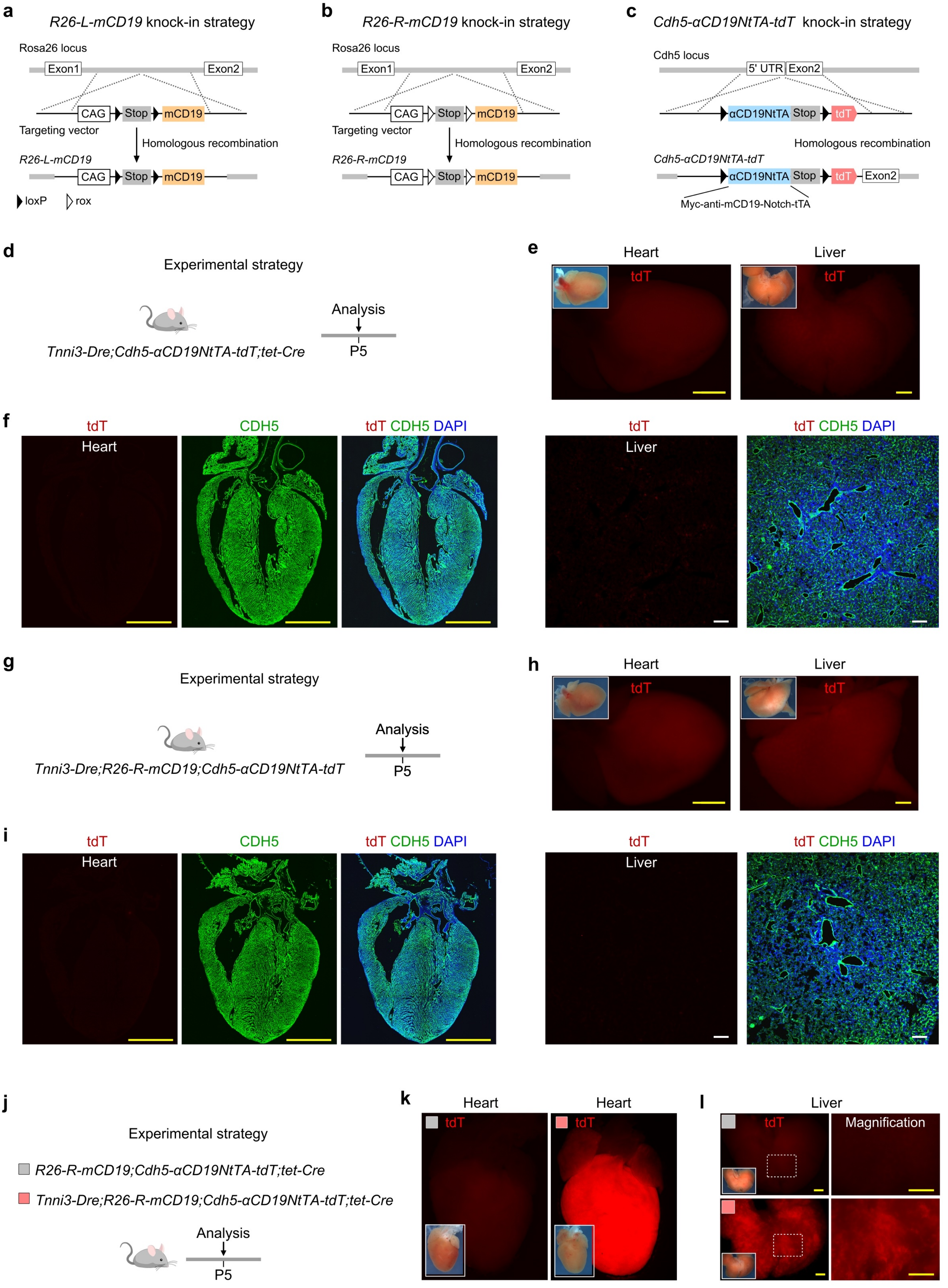

Supplementary Figure 1

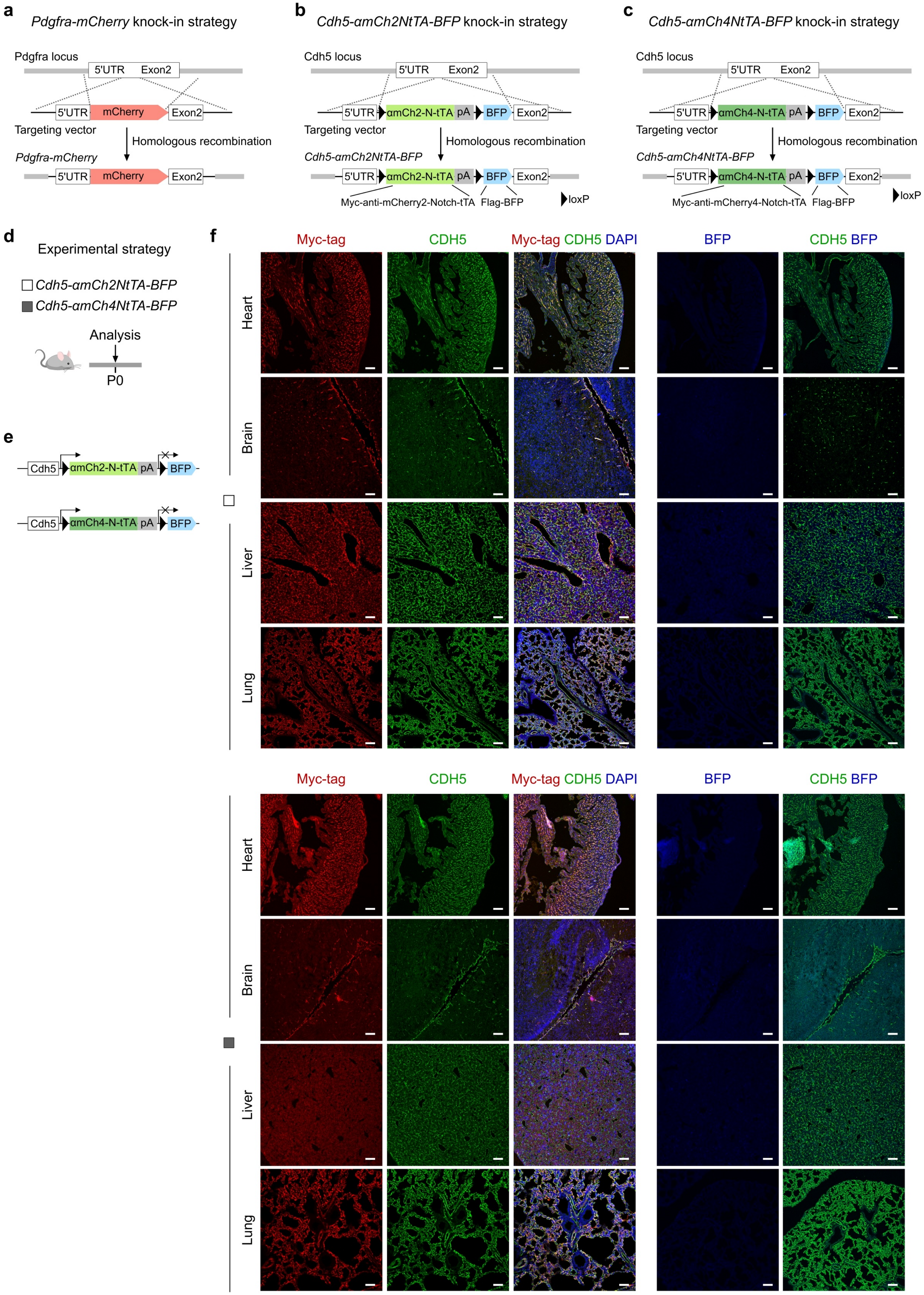

Supplementary Figure 2

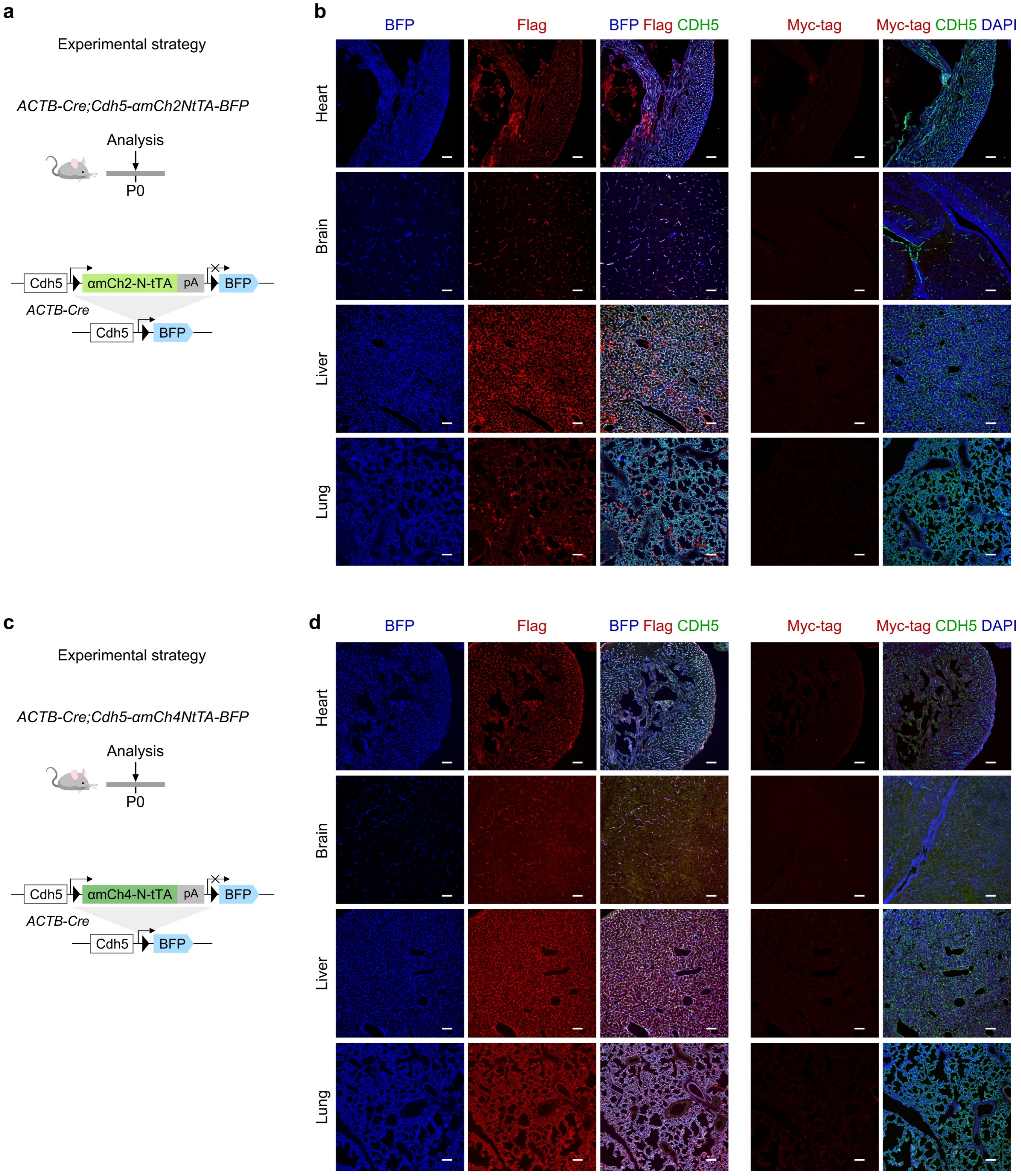

Supplementary Figure 3

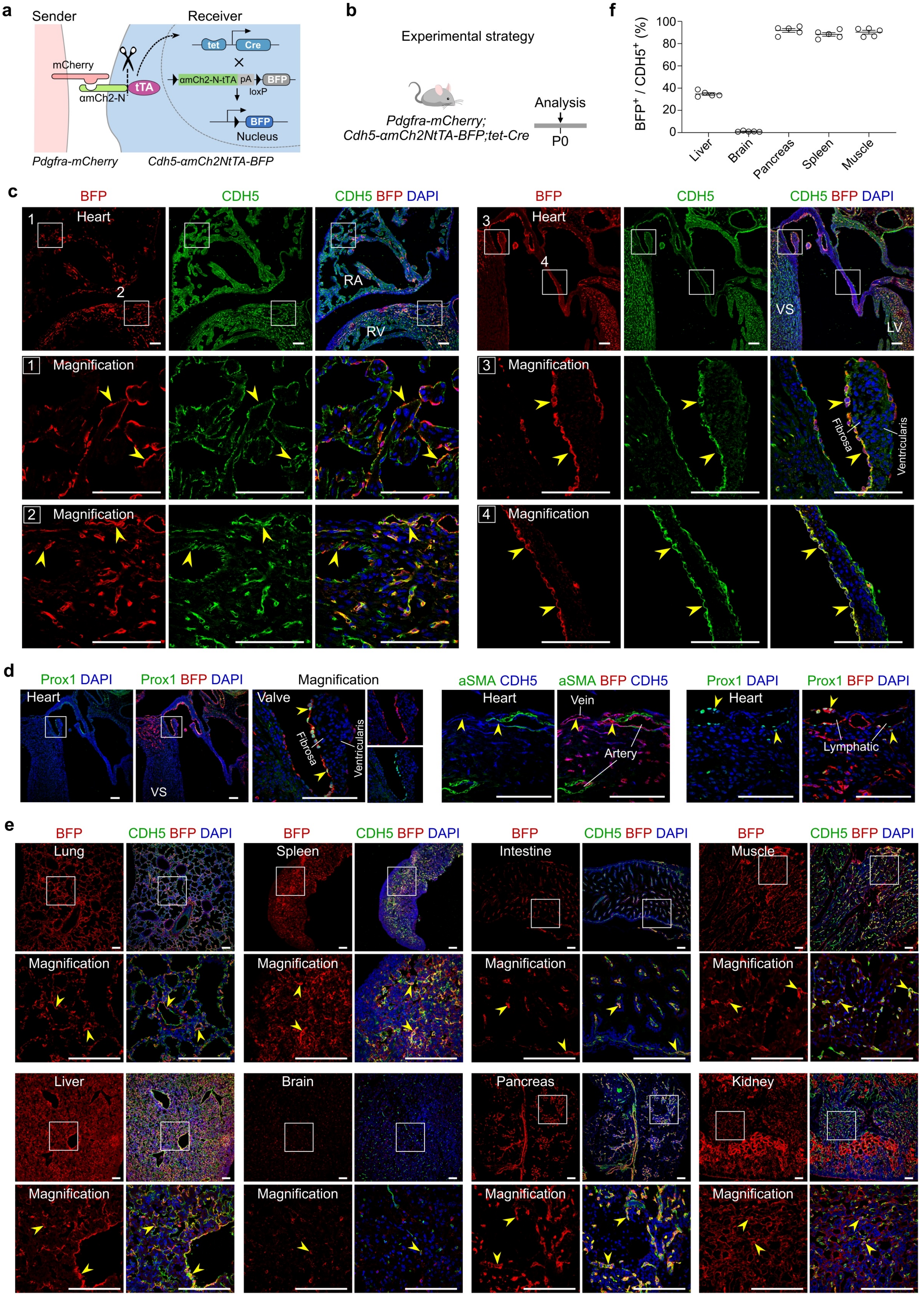

Supplementary Figure 4

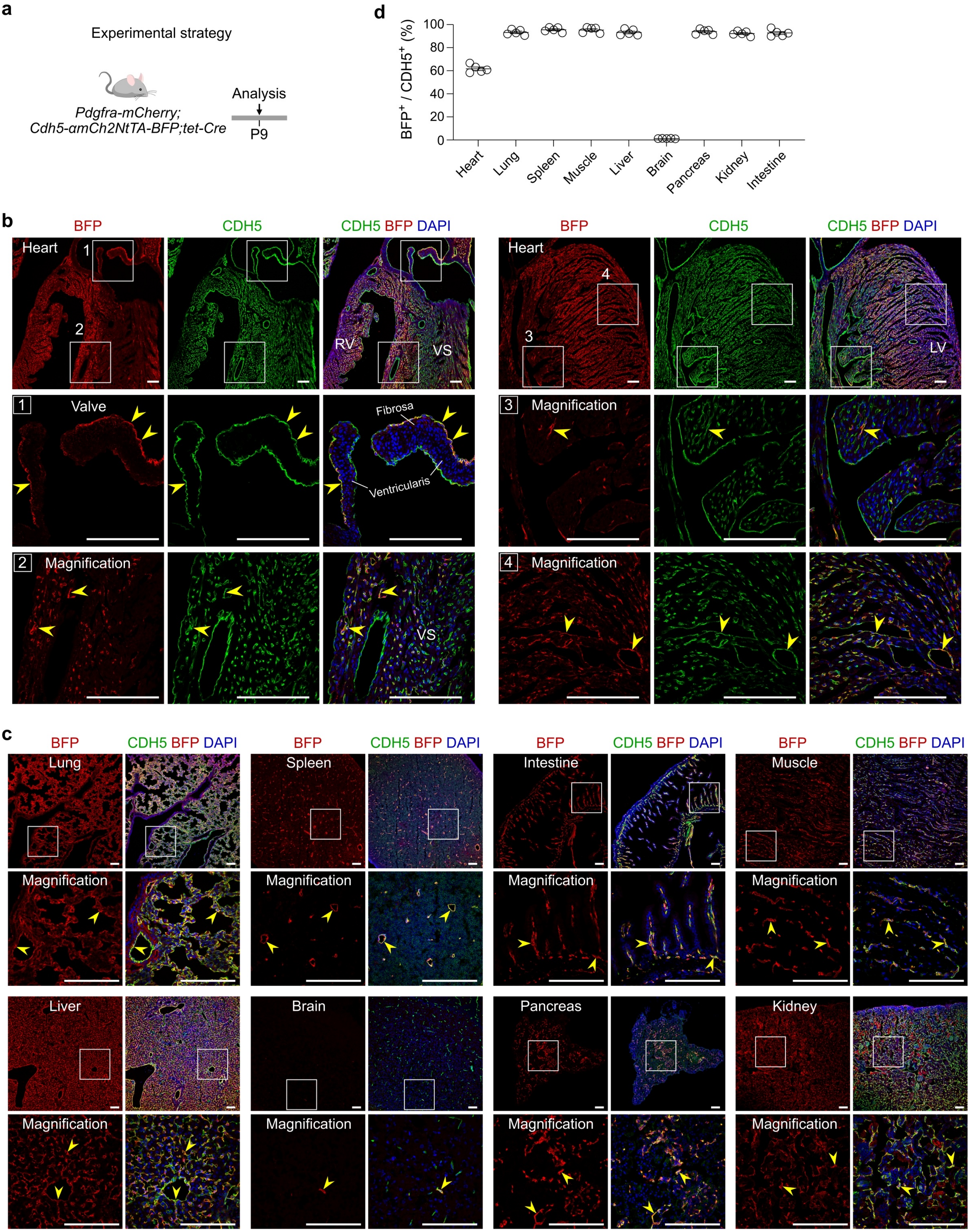

Supplementary Figure 5

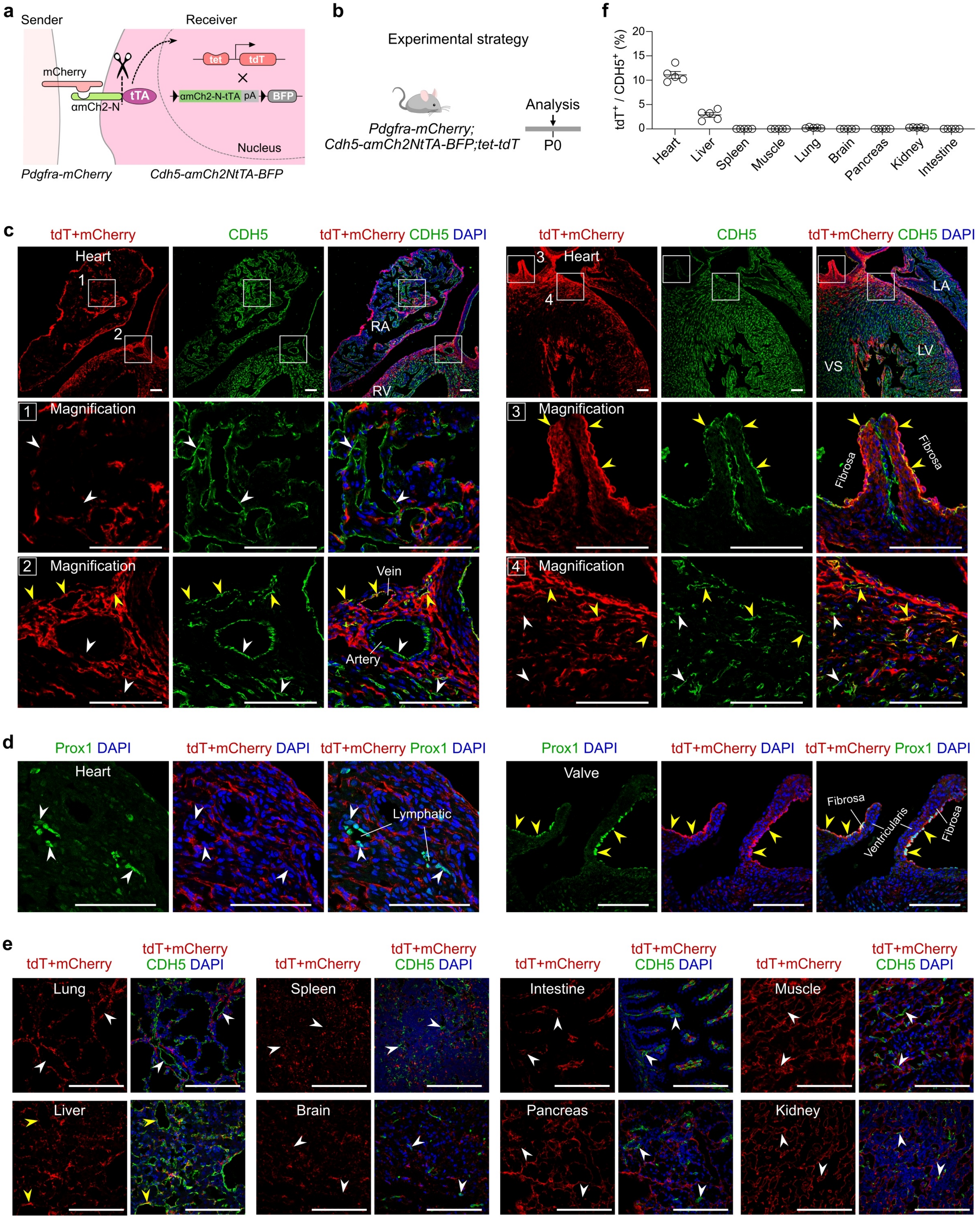

Supplementary Figure 6

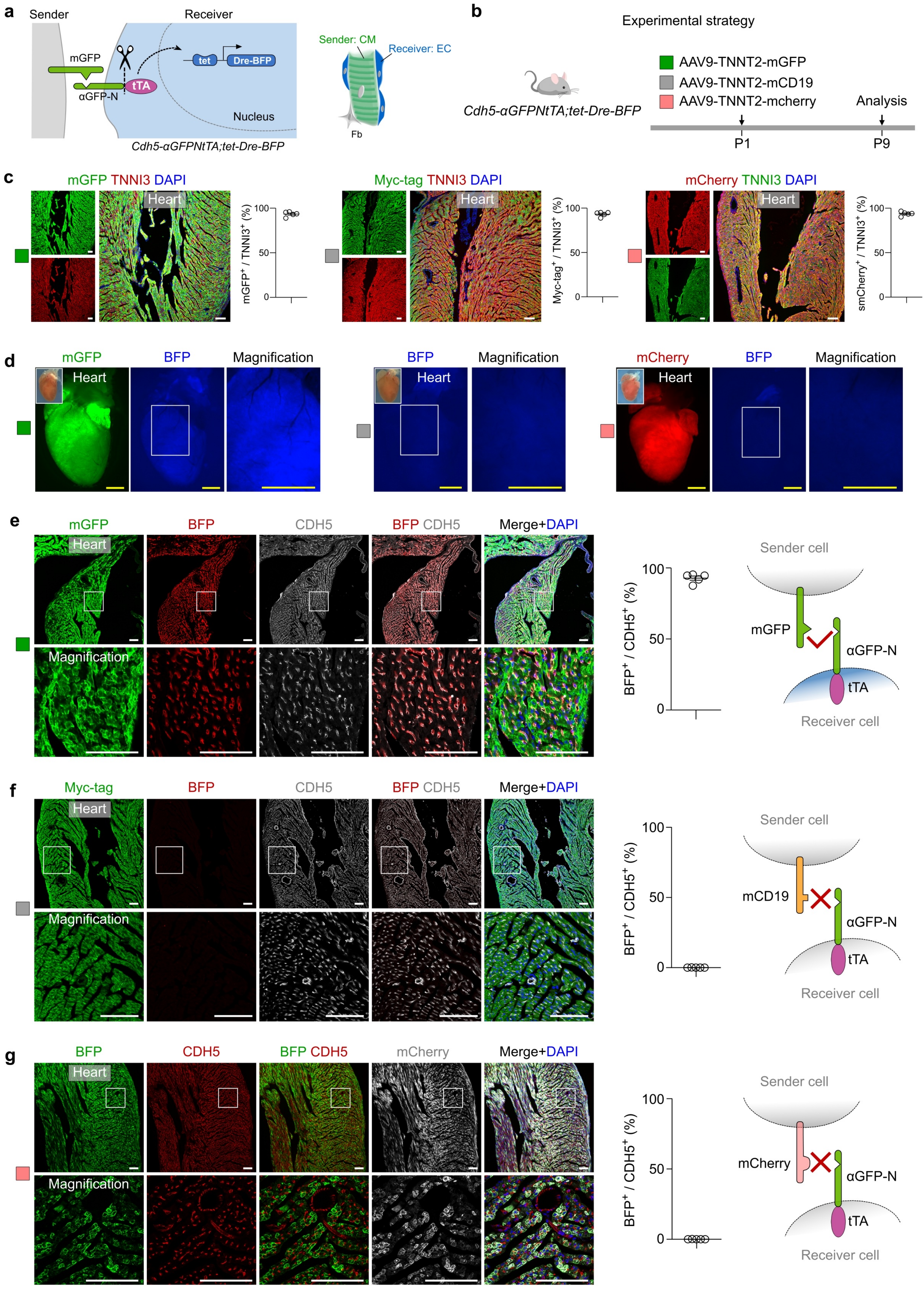

Supplementary Figure 7

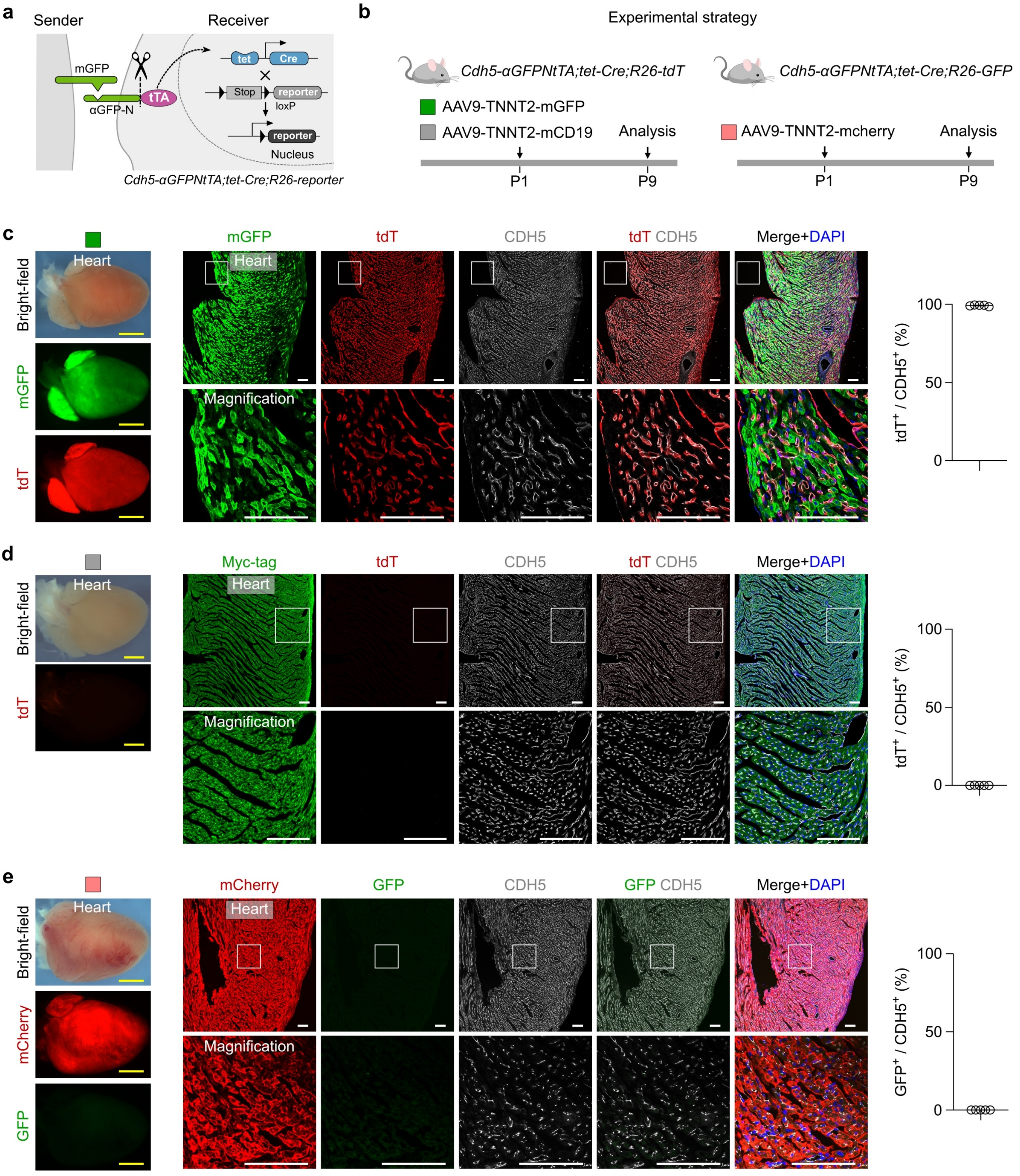

Supplementary Figure 8
